# Inventory management performance of essential medicines in public health facilities of Jimma Zone, Southwest Ethiopia

**DOI:** 10.1371/journal.pgph.0004379

**Published:** 2025-04-24

**Authors:** Azmeraw Bekele, Bekele Boche, Yeniewa Kerie Anagaw, Wondim Ayenew, Minichil Chanie Worku, Derso Teju Geremew, Tefera Minwagaw, Eneyew Melkamu, Alem Endeshaw Woldeyohanins, Bereket Bahiru Tefera, Mengistu Duguma

**Affiliations:** 1 Department of Social and Administrative Pharmacy, School of Pharmacy, Institute of Health Science, Jimma University, Jimma, Ethiopia; 2 Department of Pharmaceutical Chemistry, School of Pharmacy, College of Medicine and Health Science, University of Gondar, Gondar, Ethiopia; 3 Department of Social and Administrative Pharmacy, School of Pharmacy, College of Medicine and Health Science, University of Gondar, Gondar, Ethiopia; 4 Department of Pharmaceutics, School of Pharmacy, College of Medicine and Health Science, University of Gondar, Gondar, Ethiopia; 5 School of Pharmacy, College of Medicines and Health Sciences, Bahir Dar University, Bahir Dar, Ethiopia; 6 Department of Midwifery, College of Medicine and Health Science, University of Gondar, Gondar, Ethiopia; 7 Department of pharmacy, Seka primary hospital, Jimma zone, Jimma, Ethiopia; University of Oslo Faculty of Medicine: Universitetet i Oslo Det medisinske fakultet, NORWAY

## Abstract

The inventory management of essential medicines is a crucial aspect of health care delivery, particularly in resource-limited settings. Access to these medicines must be ensured to meet the health needs of the population, demanding effective control of their inflow, outflow, and stock levels. Poor inventory management can lead to significant disruption in the supply chain, ultimately affecting patient care and safety. The aim of this study was to assess inventory Management Performance of Essential Medicines in Public Health Facilities of Jimma Zone, Southwest Ethiopia.A facility-based descriptive cross-sectional mixed-method study was conducted using an observational checklist, semi-structured and structured questionnaires, and triangulation. Twenty public health facilities were randomly included in the study. The quantitative data were coded and analyzed using SPSS version 20. The qualitative data were analyzed using thematic analysis techniques. Out of 20 health facilities, 14 (70%) experienced stock out of 8 (66.6%) essential medicines at least once, and all health facilities experienced emergency orders of varying terms within the previous six months. The result indicated a range of reasons for stock outs of essential medicines, including insufficient medicine supply 11 (55%), stock out at re-supply point 8 (40%), drug expiration 7 (35%), and order modifications at the replenishment point 6 (30%). The mean accuracy of bin card records was 188(78.3%), whereas the mean accuracy of Report and Resupply Form reports was 87.37% with a mean discrepancy of 0.13. Of all completed Report and Resupply Form reports, 56 (93.3%) were sent on time to suppliers. Essential medicines’ inventory management performance was inadequate, characterized by prolonged stock outs, frequent emergency orders, and the need to improve storage conditions. Infrastructure-related problems, data management issues, insufficient human resource development, and the inability to integrate digital technology into the inventory management system were the key challenges identified.

## Background

Medicines are essential components of patient care, and access to essential medicines should be guaranteed [1]. The inflow and outflow of essential medicines, which is a compulsory portion of the supply chain network, must be monitored [2] and used to guard healthcare delivery against any type of disturbance [3]. Subsequently, inventory management of pharmaceuticals affects the supplies of medicine for patients and is crucial in ensuring high standards of care [4]. To this point, public health facilities rely heavily on safety stock to meet unpredictable demand, leading to high operational costs and issues with medicine expiration [5]. In contrast, this helps to maintain a steady supply to patients and prevents product stock out, while minimizing the costs of holding inventory [6]. Specially, accurate and up-to-date stock records are crucial, and should conform to standard operating procedures for enhancing inventory management performance [7]. In Sub-Saharan African countries, inventory control policies have a significant influence on the disruption of medicines, and accounting for stockouts regardless of demand seasonality and facility access interruption [8,9]. Nowadays, inventory management is aided by an electronic Logistics Management Information System (eLMIS), which allows for easier tracking of medical transactions and connects all levels of supply chains [10,11]. Evidences showed that utilization of eLMIS is increasing in many parts of Ethiopia, however, data quality and access to essential medicines were found to be inadequate, owing to poor inventory management practices throughout the public health facilities [12–14]. Hence, evaluating inventory management performance is critical to evaluate logistical activities [15], and rigorous information on the availability and quality of health services is essential for healthcare planning, management, and tracking changes over time [16]. Therefore, this study aimed to assess the inventory management performance of essential medicines in the public health facilities of Jimma Zone, Southwest Ethiopia.

## Materials and methods

### Study design, period and settings

A health facility-based descriptive mixed method cross-sectional study design was employed from December 17, 2023- January 8, 2024, in the public health facilities of Jimma Zone. It is one of the 22 zones in the Oromia region, whose administrative center is the town of Jimma, which is located 352 kilometers southwest of Ethiopia’s capital, Addis Ababa. The zone is divided into 20 districts and a two-town administration with 545 kebeles. It covers a total of 184,125.4 km2 with a total population of 3,345,112. There were 122 health facilities, 513 health posts, 8 primary hospitals, and 1 tertiary hospital in Jimma Zone [17].

### Study design

A facility-based descriptive cross-sectional mixed method study was conducted using an observational checklist, semi-structured and structured questionnaires.

### Study population and units

The study population consisted of selected public hospitals and health centers, pharmacy heads and store managers of selected health facilities, chosen essential medicines, bin cards, stock cards, and Report and Resupply Forms (RRF) reports.

### Eligibility criteria

The quantitative study was designed to include all hospitals and health centers that began their services before December 1, 2022. This ensured a representation of facilities with established operational frameworks. Additionally, the study incorporated data from RRFs and bin cards used from December 1, 2022, to December 17, 2023, providing a time-relevant data of essential medicine inventory management performances. The study also included healthcare professionals responsible for managing essential medicines, recognizing their important role and responsibility in the inventory management system. Military and missionary health facilities were excluded due to their distinct administrative platforms, management structures, and inventory management systems, which differ significantly from those of public health centers and hospitals. These differences arise from their specialized operational frameworks and unique official mandates, which are tailored to their specific contexts and populations served. Besides, respondents who refused to participate and were absent during data collection were excluded from the study.

### Sample size and sampling procedures

The sample size of the quantitative study was determined based on the logistics indicators assessment tool (LIAT) recommendation in which a minimum of 15% of the total health facilities have to be considered [18]. Health facilities were stratified by type into hospitals and health centers, with all hospitals purposively included due to their broader range of services. For health centers, a simple random sampling technique was employed: each was assigned a unique identifier, and a lottery method was used to select the sample. Accordingly, 20 health facilities were selected from the total of 134 health facilities, of which 9 were hospitals and 11 were randomly selected health centers. Twelve essential medicines and corresponding bin cards were included, with essential medicines selected based on the top 10 diseases identified by the catchment health facilities. Two of the selected medicines were used as alternative treatment. A total of six months of 60 (three RRFs from the 20 health facilities) RRF reports were reviewed. Key informants (KIs) were purposively included based on their level of responsibility in managing essential medicines until saturation of the idea was achieved. Each health facility was chosen at random using a lottery method from each district that did not contain a hospital. However, all hospitals were included because they were located in different district.

### Data collection tool

The data collection tools were adopted from the Logistics System Assessment tool (LSAT), Inventory Management Assessment Tool (IMAT) and LIAT developed by USAID/DELIVER Project [18–20].

### Data collection procedure

The data were gathered using a self-administered questionnaire, filling out checklists based on physical observation and physical count of medicines. Bin card/stock cards, and RRF reports were reviewed form December 18, 2023 to January 2, 2024. Three pharmacists collected relevant data after receiving half-day training on the study objectives, technical terms, and a written guide along with the questionnaires. The qualitative data was gathered through in-depth face-to-face interviews with KIs such as facility directors, pharmacy heads and store managers using an interview guide. Each KI was interviewed and recorded for 15–20 minutes.

### Data management, and analysis

The quantitative data was validated for completeness, entered into an MS Excel 2016 spreadsheet, and then exported to the Statistical Package for Social Sciences (SPSS version 20) for analysis. The results were presented using, texts, tables and graphs. The qualitative data were manually analyzed using thematic analysis techniques. Thus, the investigators became acquainted with the recorded data by listening repeatedly and taking notes. Investigators then coded the data using a Microsoft Excel tabular table. The coded data were grouped to facilitate the search for subthemes. Similar subthemes were combined to create a theme. Lastly, we used themes and direct quotes from KIs to boost our description.

### Operational definition of terms

The performance was measured using specific indicators because it is difficult to measure overall inventory management performance from poor to excellent [21] (S1 Table).

#### Essential medicines.

: Essential medicines are those that satisfy the priority health care needs of a population [1]

#### Acceptable storage conditions.

: A health facility’s storage condition is considered acceptable if it meets at least 80% of the criteria for appropriate storage conditions [22]

#### Stock wastage.

: The acceptable standard for unusable items is <2% of total item value [22]

#### Data quality of RRF reports.

: It is the measurement of data accuracy, completeness, timeliness, and facility reporting rate [23].

#### Completeness of reports.

: a report is considered complete if all the columns for each product listed in the report are filled unless the facility does not manage the product [23]

#### Data accuracy.

: Data is considered accurate when there is no discrepancy between stock balances on the bin card record (manual, electronic) compared with the physical count and the balance on the bin card to the balance on the RRF report: inventory accuracy was good if (≥80%) and vice versa [22]

#### Timelines of reports.

as per the standard operating procedure of the integrated pharmaceutical logistics system (IPLS) of Ethiopia [24]

I. Hospitals and health facilities should submit their RRF reports to the Ethiopian Pharmaceutical Agency or the Zone Health Department by the 10th day of the third month [25].
II. Health centers that provide their RRF reports through the district health office should submit them by the 5th day of the third month.

#### Bin card updated.

: if the bin card was last updated with a balance of zero and the facility has not received any of those products within the previous 30 days [23]

## Results

### Socio-demographic characteristics of participants

The study included 80 health facility staff, of which 55 were pharmacy professionals and 25 were other health professionals. Only five (6.25%) of the total staff held a second degree. The majority of the participants were males 61 (76.25%), and 20 (25%) had IPLS training through various courses (Table 1).

**Table 1:** Socio-demographic backgrounds of participants in selected public health facilities of Jimma zone, 2024.

Variables			Percentage n (%)
Profession	Pharmacy		55(68.75)
	Other health		25(31.25)
Sex	Male		61(76.25)
	Female		19(23.75)
Service year	<1		26(32.5)
	1-5		34(42.5)
	>5		20(25)
Level of Education	1^st^ degree (pharmacy)		35(43.75)
	Diploma	Nurse to pharmacy	20(25)
		Nurse	20(25)
	2^nd^ degree health professionals		5(6.25)
Received LMIS training	During IPLS training		16(20)
	During a logistics workshop		2(2.5)
	On-the-job training		2(2.5)

### Availability and updating practice of bin card records

All health facilities managed selected essential medicines with all bin card records available for oxytocin injection, Artemether-lumefantrine, rifampicin–isoniazid–pyrazinamide–ethambutol combination for treatment of Tuberculosis (RHZE Tb) kit, Amoxicillin 500mg capsule, and Sulfamethoxazole-trimethoprim240mg/5ml solution. Most health facilities updated their bin card records for all essential medicines, although there was a drug like Amoxicillin 500 mg capsule with the least updated bin card records (Table 2).

**Table 2:** Availability and updating practice of bin card records in selected public health facilities of Jimma zone, 2024.

S/No	List of Essential medicines	Unit	Managed at this facility	Bin-card available		Bin card updated	
			Yes (%)	Yes (%)	No(%)	Yes(%)	No (%)
1	Amoxicillin 250 mg dispersible tablet	Tab	20(100)	17(85)	3(15)	13(65)	7(35)
2	ORS	Sachet	20(100)	18(90)	2(10)	12(60)	8(40)
3	Tetracycline 4g eye ointment	4gm	20(100)	18(90)	2(10)	14(70)	6(30)
4	Oxytocin injection	Amp	20(100)	20(100)	–	15(75)	5(25)
5	Artemether-Lumefantrine	Tab	20(100)	20(100)	–	13(65)	7(35)
6	RHZE (Tb kit)	Kit	20(100)	20(100)	–	16(80)	4(20)
7	Ciprofloxacin 500 mg tablet	Tab	20(100)	17(85)	3(15)	12(60)	8(40)
8	Amoxicillin 500 mg capsule	Caps	20(100)	20(100)	–	18(90)	2(10)
9	Sulfamethoxazole trimethoprim240mg/5ml Suspension	100ml	20(100)	20(100)	–	13(65)	7(35)
10	Diclofenac 75mg/5ml injection	Amp	20(100)	17(85)	3(15)	14(70)	6(30)
11	Mebendazole 200 mg tablet	Tab	20(100)	17(85)	3(15)	15(75)	5(25)
12	Doxycycline 100mg	Caps	20(100)	19(95)	1(5)	13(65)	7(35)

### Accuracy of bin card records

The reported average bin card accuracy was 188 (78.3%); a promising level of record-keeping compliance with different accuracy among medicines. For instance, Tetracycline 4g eye ointment had higher record accuracy (95%) than Doxycycline 100mg capsule (60%) (Table 3).

**Table 3:** Accuracy of bin card records for essential medicines in selected public health facilities of Jimma zone, 2024.

S/No	List of Essential medicines	Issuing Unit	Accurate bin card N (%)
1	Amoxicillin 250 mg dispersible tablet	Tab	18(90)
2	ORS	Sachet	13 (65)
3	Tetracycline 4g eye ointment	4gm	19(95)
4	Oxytocin injection	Amp	14(70)
5	Artemether-Lumefantrine	Tab	16(80)
6	RHZE (Tb kit)	Kit	13(65)
7	Ciprofloxacin 500 mg tablet	Tab	17(85)
8	Amoxicillin 500 mg capsule	Caps	16(80)
9	Sulfamethoxazole trimethoprim240mg/5ml Suspension	100ml	17(85)
10	Diclofenac 75mg/5ml injection	Amp	18(90)
11	Mebendazole 200 mg tablet	Tab	15(75)
12	Doxycycline 100mg	Caps	12(60)
Average accuracy			188(78.3)

*RHZE: rifampicin–isoniazid–pyrazinamide–ethambutol; ORS: Oral Rehydration Solution.*

### Data transferring accuracy of RRF REPORTS

The average accuracy of RRF reports was 87.37% with a mean discrepancy of 0.13 and the highest discrepancy seen for Artemether-Lumefantrine (13.24%). Most of the RRF reports did not encounter any discrepancies in the transfer of data from the bin card to the RRF report (Table 4).

**Table 4:** Data transferring accuracy of RRF reports in selected public health facilities of Jimma zone, 2024.

Product	Usable Stock on Hand		Discrepancy
	From a recent RRF report (A)	From bin or stock cards (B)	
Amoxicillin 250 mg dispersible tablet	396000tab	396000tabs	0
Oral Rehydration salt ORS	547655sachet	547655sachet	0
Tetracycline 4g eye ointment	10000tube	11000tube	9.1
Oxytocin injection	118000amp	118700amp	0.59
Artemether-Lumefantrine	59982strip	68982strip	13.24
RHZE (Tb kit)	13200kit	13200kit	0
Ciprofloxacin 500 mg tablet	643473strip	660473strip	2.58
Amoxicillin 500 mg capsule	233600strip	234100strip	0.21
Sulfamethoxazole-trimethoprim240mg/5ml suspension	83698bot	83698bot	0
Diclofenac75mg/5ml injection	132078amp	132078amp	0
Mebendazole 200 mg tablet	220567strip	220567strip	0
Doxycycline 100mg	440556strip	440556strip	0
Average discrepancy in transferring information to the RRF reports			12.63
% Accuracy			
Average accuracy of transferring information to the RRF reports			87.37

*RHZE: rifampicin–isoniazid–pyrazinamide–ethambutol; ORS: Oral Rehydration Solution.*

### Essential data components of LMIS records and reports

The three essential data parts of LMIS for bin card records were filled in 17 (85%) health facilities (Table 5). It is required that the three essential LMIS data such as stock on hand, consumption, losses, and adjustments must be filled in all LMIS records and reports.

**Table 5:** Filled essential data components of LMIS records and reports in selected public health facilities of Jimma zone, 2024.

		Yes (%)	No (%)
Stock card		9(45)	11(55)
Bin card	All data points	17(85)	3(15)
	Stock on hand	20(100)	0(0)
	Consumption	20(100)	0(0)
	Losses and adjustments	7(35)	13(65)
RRF ^*^		20(100)	0(0)
	All data points	6(30)	14(70)
Daily Register		8(40)	12(60)

*RRF: report and resupply form;*

*Stock on hand, consumption, losses and adjustment.

### Timeliness and completeness of RRF Reports

**Fig 1:**
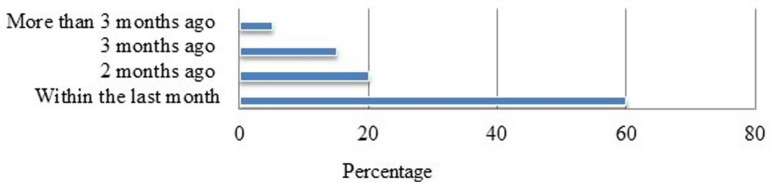
Facilities reporting time for product resupply in selected public health facilities of Jimma Zone, 2024.

Most of health facilities send their RRF reports in the last month of the data collection period. On the other hand, on average, 56 (93.3%) of RRF reports were submitted on time and found complete (Fig 1).

### Emergency order placed by the health facilities

All health facilities experienced emergency orders of varying terms within the previous six months. The majority, 9 (45%), of them placed more than 5 emergency orders and the remaining facilities encountered 1–3 emergency orders. The quantity of demand for essential medicines was determined by health facilities, suppliers, and higher-level management accounted for 17 (85%), 2 (10%), and 1 (5%), respectively.

### Stock status of essential medicines

Fourteen (70%) health facilities were stocked out of essential medicines for various periods. Twelve (60%) health facilities experienced stock out of Artemether-lumefantrine while overstocked for mebendazole prior to the resupplying period (Table 6).

**Table 6:** The stock status of essential medicines during the facility visit in selected public health facilities of Jimma Zone, 2024.

S/No	Status/Product Items	Percentage
1	**Stock out items before resupplying**	
1.1	Artemether-lumefantrine	12(60)
1.2	Amoxicillin 250mg tab	12(60)
1.3	Cotrimoxazole syrup	10(50)
1.4	Amoxicillin	8(40)
1.5	Ciprofloxacin 500mg	8(40)
2	**Overstock items before resupplying**	
2.1	Mebendazole tab	9(45)
2.2	Oxytocin injection	8(40)
2.3	Zink-ORS copeck	6(30)

### Stock out of essential medicines

Eight (66.6%) of the essential medicines were stocked out at least once within the last six months and Artemether-Lumefantrine combination were out of stock for an extended duration (Table 7).

**Table 7:** Stock out rate key essential medicines in the last six months in selected health facilities of Jimma Zone, 2024.

List of essential medicines	X̄ stock out frequency in months	X̄ stock out duration in days	Avg. % of stock out
Amoxicillin 250 mg dispersible tablet	1	34.24	
Tetracycline 4g eye ointment	1.03	42.51	
Artemether-Lumefantrine	1.57	43.61	
Ciprofloxacin 500 mg tablet	1.32	32.45	8.33
Amoxicillin 500 mg capsule	1.13	23.52	
Sulfamethoxazole-trimethoprim 240mg/5mlsuspension	1.26	29.53	
Diclofenac75mg/5ml injection	1.41	37.43	
Doxycycline 100mg	1.37	28.41	

### Reasons for stock out

The findings revealed a variety of reasons for stock outs of essential medicines, such as inadequate medicine supply 11(55%), stock out at re-supply point 8(40%), drug expiration 7(35%), and order changes at the replenishment point 6(30%). Stock out at the re-supply point was identified as the most common cause for Artemether-Lumefantrine 14(70%) and Diclofenac injection 10(50%). The main reason for stockouts of Sulphametoxazole-trimethoprim240mg/5ml suspension, Ciprofloxacin 500 mg tablet, Doxycycline 100mg, and Tetracycline 4gm eye ointment, among others, was a lack of adequate supply of medicines from the re-supply point.

### Storage conditions of the facilities

The average acceptable storage status was 81.2 percent. Availability and accessibility of fire safety equipment were identified as the least fulfilled criteria for storage conditions. Another disappointing issue is that only 9 (45%) of the facilities had adequate room for drug storage and free space for future expansion (Table 8).

**Table 8:** Storage conditions of selected essential medicines in selected health facilities of Jimma Zone, 2024.

No.	Description	Yes N (%)
**1.**	Products that are ready for distribution are arranged so that identification labels expiry dates and/or manufacturing dates are visible.	18 (90)
**2.**	Products are stored and organized in a manner accessible for first-to-expire, first-out (FEFO) counting and general management.	8 (40)
**3.**	Cartons and products are in good condition, not crushed due to mishandling.	16(80)
**4.**	The facility makes it a practice to separate damaged and/or expired products from usable products and remove them from inventory.	17(85)
**5.**	Products are protected from direct sunlight at all times of the day	19(95)
**6.**	Cartons and products are protected from water and humidity	20(100)
**7.**	The storage area is visually free from harmful insects and rodents	19(95)
**8.**	Storage area is secured with a lock and key, but is accessible during normal working hours; access is limited to authorized personnel.	15(75)
**9.**	Products are stored at the appropriate temperature during all seasons according to product temperature specifications	20(100)
**10.**	The roof is always maintained in good condition to avoid sunlight and water	20(100)
**11.**	The storeroom is maintained in good condition	17(85)
**12.**	The current space and organization are sufficient for existing products and reasonable expansion	9(45)
**13.**	Products are stacked at least 10 cm off the floor.	18(90)
**14.**	Products are stacked at least 30 cm away from the walls and other stacks.	15(75)
**15.**	Products are stacked no more than 2.5 meters high.	20(100)
**16.**	Fire safety equipment is available and accessible	5(25)
**17.**	Products are stored separately from insecticides and chemicals	20(100)
	Average	276(81.2)

### Inventory management performance challenges of essential medicines

A total of 15 KIs were interviewed regarding the challenges of managing essential medicines, and the results were thematically organized into the following themes.

### Human resources development-related challenges

The majority of KIs in most health facilities complained about restricted training opportunities, high workloads due to staff shortages, inadequate support for professional development, insufficient budgets, and limited access to essential resources. This can be exemplified by a health center store manager as follows:

*“I can verify that several of my colleagues have voiced their concerns about their terrible experience with a high workload due to staff shortages, as well as the abused bureaucratic approach to recruiting trainees. Sometimes one person receives repeated training while others do not in the same field of practice, which ultimately hinders our interest in providing quality care. I can tell you that no one is interested in reading standards and the IPLS SOP guide because we believe the leadership process is unjust and determined by the administrative interest rather than the requirement of professional situations.”*

Most of the KIs believed that the administrators were confounded by the number of employees necessary for the job and their associated salaries; therefore, the administration focused more on cost savings. One of the hospital directors replied that:

“*Our focus has increasingly shifted towards cost-saving measures, often at the expense of adequate staffing and support for our teams*. *Due to budget restrictions, our employees may have had limited access to ongoing professional development and training, resulting in knowledge gaps. Economic pressures across the country frequently result in understaffing, which leads to an overload for our employees.*”

### Data management and digital technology integration-related challenges

As per the KI responses, almost two-thirds of health facilities formally monitored their LMIS records and reports before and after the reporting periods. LMIS tools were inadequately recorded and updated for each pharmaceutical stocking and transaction, resulting in increased customer dissatisfaction with product stock outs and unnecessary waste of essential medicines. Several health facilities struggled to adapt to new policy standards, resulting in inconsistencies in inventory management systems. A hospital pharmacy head replied the following:

“*We have faced resistance from our staff to implementing new practices and technologies due to a lack of training and concern about additional duty. This is aggravated by the lack of budget allocated for the training and unfailing use of digital tools like DAGU 2.1 to support inventory management system.*”

On the other hand, more than half of KIs rejected the need for additional funds for training and the expansion of digital tools, mentioning a culture of competition and negligence among healthcare staff that resulted in poor inventory exchange. The majority of the health facilities were not effective in using inventory management techniques such as Always Better Control (ABC) analysis, Vital, Essential and Non-Essential (VEN) medicines analysis and VEN/ABC matrix to ensure the availability of affordable essential medicines. A health center pharmacy head explained their working practice as follows:

“*Despite the possible advantages of the effective use of inventory management techniques, we are ruled by a cycle of irregular practices. Our facility has a common culture of prioritizing immediate cost savings over long-term investments in training and technology. As a result, we frequently find ourselves with either an excess of non-essential items or frequent shortages of inexpensive essential medicines*.”

### Infrastructure and storage optimization-related challenges

Most KIs appreciate that clients get early and complete service when they order from a well-run store; however, they complained that the health facilities faced a shortage of space and store handling equipment. As a result, they struggled to implement the FEFO technique for stock rotation. Stored on pallets, some of the products have made physical tracing difficult, and some of them, although expired, were not identified. A hospital store man stated that:

“*We lack a suitable ventilated store and several medicines are in unsafe conditions. They are stored on pallets or in tight spaces. This setup makes it hard to manage proper stock rotation. We often have many expired medications that go unreported. While our service delivery performance has not only been affected by this situation, it has also raised serious concerns about the safety of our patients. During my five years as a store manager, the administration often treated pharmaceuticals as regular items for sale.”*

As responded by KIs, the health centers and a hospital lacked adequate space for the volume of inventory they needed to manage, climate control, and stock tracking technology. Many health facilities lacked temperature monitoring instruments, while others had non-calibrated instruments. The absence of a humidity monitoring instrument was a prevalent concern in all health facilities.

## Discussion

Well-organized inventory management of essential medicines is critical for operational effectiveness, improving patient care, and guaranteeing the sustainability of healthcare delivery systems [26,27]. The findings of this study deal with product stock status, product waste, storage conditions, and key challenges related to the inventory management performance of essential medicines. Thus, the current study showed that the practice of updating stock-keeping records was uneven; for example, Amoxicillin 500 mg capsule had the most updated bin card records, while ORS and Ciprofloxacin 500 mg tablet had the least, raising concerns for inventory accuracy and availability and many KIs were complaining for high workloads and inadequate staff training as barriers to on-time practice.

The current study found that the average accuracy of recording counted essential medicines on the bin card was 78.3%, with Tetracycline 4g eye ointment attaining the maximum accuracy of 95% and Doxycycline 100 mg capsules recording the lowest of 60%. This indicates that approximately one in every five records could be inaccurate, perhaps resulting in medication errors or supply disparities. If records don’t reflect the actual quantity or strength of medicines available, healthcare providers might administer incorrect dosages, either too high or too low, compromising patient safety. Repeated errors due to logistical issues can erode patient trust in the healthcare system, leading to non-adherence and worse health outcomes. Variations in inventory management performance among different medicines may be due to issues related to storage conditions, stock tracking technology, and a lack of staff training for the appropriate positions, as revealed by the qualitative findings. The average accuracy of stock-keeping records ranged from 30.4% to 78.5% across studies [13,28,29].

The inconsistency between the studies could be attributed to differences in counting and recording practices or a greater range of drugs with different handling and storage requirements. Seven (58.33%) of the essential medicines were available during the facility visit, with 3 (25%) being overstocked. This suggests that nearly 42% of essential medicines were unavailable, potentially leading to gaps in patient treatment options; nonetheless, overstocking can result in a waste of medicines, even though these drugs were identified as essential medicines that are always available. The current study’s low availability of essential medicines, 58.33%, as compared to other similar studies with overall availabilities of 74.7% [30], and 78.6% [31] could be due to the larger sample size in the current study that may include a large number of low-performing health facilities.

This study also found that 70% of health facilities suffered stock outs, with a mean stock-out duration of 33.69 days and an average daily stock out of 8.33% within the last six months, showing flaws in supply chain management, which is in line with a previous similar study with specific elements, where 69.6% of the health facilities experienced at least one stock out with an average daily stock out of 4% [32] but lower than other similar studies with mean stock-out durations of 70.71 days [13], and 38.8 days [33]. The longer stock-out durations in the previous studies could be related to the larger number of essential medicines reviewed compared to the current study, which increases the possibility of extended stock-outs.

This study also revealed that the average data accuracy of RRF reports was 87.37%, indicating significant discrepancies in data transferring accuracy from bin cards to RRF reports. This study’s KIs also noticed that the LMIS tools were not properly recorded or updated, resulting in increased stock outs and waste of essential medicines contributing to consumer discontent. The current value is higher than similar previous studies with accurate RRF reports of 59.48% [13], and 72.2% [34]. The difference could be related to a difference in the number of medicines reviewed, as the previous studies considered additional medicines than the current study, which may have increased the possibility of information transfer inaccuracies.

The current study evaluated the completeness and timeliness of the RRF reports and came up with an equivalent result of 93.3%. This demonstrates that the framework for producing RRF reports is reliable, and allows for quick responses. The present study identified more complete and on-time RRF reports than in prior studies of similar interests with 88.8% [34], 40.52% [13], 66.67% [35] on time, and 75% [34], 62.93% [13] complete reports. The large disparity across the studies could be attributable to differences in sample size and reporting time, indicating a lack of consistent use of the same standard operating procedures set by the National Ministry of Health.

Although this study provides useful insights into inventory management practices, there are areas for improvement in future research attempts to provide more robust and comprehensive results. Thus, this study has the following limitations: the indicators used may not cover all relevant aspects of inventory management performances, and the sample size is not large enough, which may affect the generalizability of the findings to wide range of large populations.

## Conclusion

In conclusion, the inventory management performance was inadequate, characterized by prolonged stock outs, frequent emergency orders, and the need to improve storage conditions. The study also identified challenges related to infrastructure, storage optimization, data management, digital technology integration, and human resource development. To address the above identified problems, health authorities must implement interventions that focus on designing targeted training programs for healthcare professionals, improving the effectiveness and usability of digital inventory management system means to streamline inventory tracking and reporting, and establishing a regular feedback mechanism to monitor inventory levels and address stock-out difficulties.

## Supporting information

S1 Table Indicators used to measure inventory management performance (20–22,35). (DOCX)
