# Supplementary material for: Inventory management performance of essential medicines in public health facilities of Jimma Zone, Southwest Ethiopia
Source: PLOS Glob Public Health. 2025 Apr 24;5(4):e0004379. doi: 10.1371/journal.pgph.0004379 (PMC12021134; doi:10.1371/journal.pgph.0004379)
Supplement: S1 Table — (DOCX) [file pgph.0004379.s001.docx]

S1 Table. Indicators used to measure inventory management performance (20–22,35)

| Indicators (%) | Measurement Formula | Ideal |
| --- | --- | --- |
| Avg. % of stock out | $\frac{Days out of stock}{Total number of products\times number of days}\times100$ | 0 |
| Stock Wasted | $\frac{Unusable stock}{Total quantity of usable and Unusable stock}\times100$ | < 2(%) |
| Unusable stock because of damage | $\frac{unusable physical stock count attributable to damage}{total unusable physical stock count}\times100$ | TBD* |
| Value of unusable stock | $\frac{value of unusable physical stock}{value of commodity purchases}x100$ | TBD* |
| Accuracy stock records | $\frac{stock record count-physical stock count}{physical stock count}x100$ | 100% |
| Accuracy of LMIS reports | $\frac{LMIS record count - stock record count}{stock record count}\times100$ | 100% |
| Products  available | $\frac{Total products in stock}{Total number of products in the study}x100$ | 95-100% |
| Timeliness of reports | $\frac{total number of reports recieved ontime}{Total No.reports expected*}\times100$  *No. of health facilities that should report× Number of RRF reports expected from each facility | TBD* |
| Health facility reporting rate | $\frac{number facilities submitted a report at scheduled time}{Total No. of facilities required to report}\times100$ | TBD* |
|  |  | TBD*; to be determined |
